# New York Medical Times

**Published:** 1852-10

**Authors:** 


*New York Medical Times.*—Dr. Adams has resigned, on account of ill health, the editorial charge of this Journal, and is succeeded by Dr. Bulkley. Dr. Bulkley is known to the medical public as an able writer and teacher. We need not state that he is particularly distinguished for his attainments in the province of dermatology, our readers doubtless being familiar with his valuable contributions to the literature of that subject. We cordially wish him success in his present undertaking.

